# Comparative study of the protein profiles of Sunki mandarin and Rangpur lime plants in response to water deficit

**DOI:** 10.1186/s12870-015-0416-6

**Published:** 2015-03-03

**Authors:** Tahise M Oliveira, Fernanda R da Silva, Diego Bonatto, Diana M Neves, Raphael Morillon, Bianca E Maserti, Mauricio A Coelho Filho, Marcio GC Costa, Carlos P Pirovani, Abelmon S Gesteira

**Affiliations:** Universidade Estadual de Santa Cruz-UESC, Rodovia Ilhéus-Itabuna, Km 16, Salobrinho, Bahia Brazil; Centro de Biotecnologia, Departamento de Biologia Molecular e Biotecnologia, Universidade Federal do Rio Grande do Sul-UFGRS, Avenida Bento Goncalves, 9500 Porto Alegre, Rio Grande do Sul Brazil; IVIA; Centro de Genomica, Ctra. Moncada-Náquera Km 5, 46113 Moncada, Valencia Spain; CIRAD, UMR AGAP, Avenue Agropolis - TA A-75/02 – 34398, Montpellier Cedex 5, France; Dipartimento di Scienze BioAgroAlimentari, CNR-IPSP, Istituto per la Protezione Sostenibile delle Piante, Area della Ricerca CNR, Via Madonna del Piano 10, Via Madonna del Piano n 10, 50019 Sesto Fiorentino, FI Italy; Embrapa Mandioca e Fruticultura, Rua Embrapa, s/n, Cruz das Almas, 44380-000 Bahia Brazil

**Keywords:** Citrus rootstock, Water deficit, Proteomics, Protein network

## Abstract

**Background:**

Rootstocks play a major role in the tolerance of citrus plants to water deficit by controlling and adjusting the water supply to meet the transpiration demand of the shoots. Alterations in protein abundance in citrus roots are crucial for plant adaptation to water deficit. We performed two-dimensional electrophoresis (2-DE) separation followed by LC/MS/MS to assess the proteome responses of the roots of two citrus rootstocks, Rangpur lime (*Citrus limonia* Osbeck) and ‘Sunki Maravilha’ (*Citrus sunki*) mandarin, which show contrasting tolerances to water deficits at the physiological and molecular levels.

**Results:**

Changes in the abundance of 36 and 38 proteins in Rangpur lime and ‘Sunki Maravilha’ mandarin, respectively, were observed via LC/MS/MS in response to water deficit. Multivariate principal component analysis (PCA) of the data revealed major changes in the protein profile of ‘Sunki Maravilha’ in response to water deficit. Additionally, proteomics and systems biology analyses allowed for the general elucidation of the major mechanisms associated with the differential responses to water deficit of both varieties. The defense mechanisms of Rangpur lime included changes in the metabolism of carbohydrates and amino acids as well as in the activation of reactive oxygen species (ROS) detoxification and in the levels of proteins involved in water stress defense. In contrast, the adaptation of ‘Sunki Maravilha’ to stress was aided by the activation of DNA repair and processing proteins.

**Conclusions:**

Our study reveals that the levels of a number of proteins involved in various cellular pathways are affected during water deficit in the roots of citrus plants. The results show that acclimatization to water deficit involves specific responses in Rangpur lime and ‘Sunki Maravilha’ mandarin. This study provides insights into the effects of drought on the abundance of proteins in the roots of two varieties of citrus rootstocks. In addition, this work allows for a better understanding of the molecular basis of the response to water deficit in citrus. Further analysis is needed to elucidate the behaviors of the key target proteins involved in this response.

**Electronic supplementary material:**

The online version of this article (doi:10.1186/s12870-015-0416-6) contains supplementary material, which is available to authorized users.

## Background

Among potential abiotic stresses, water deficit is considered to have the largest effect on agricultural productivity and is one of the main factors limiting the distribution of species worldwide [1]. When plants are subjected to water deficit, numerous morphological and physiological responses are observed, and the amplitude of these responses depends on the plant genotype as well as the duration and severity of the stress [2,3].

The plant response to water deficit involves several processes, beginning with the perception of stress, followed by modulation of the expression of specific genes, and finally, the appearance numerous transcriptomic, proteomic and metabolomic changes. These changes result in the regulation of metabolism and the generation of regulatory networks that are involved in plant defense against the harmful effects of stress [4,5].

Transcriptomic studies have revealed that the expression of a wide range of genes is regulated in response to water deficit in citrus plants. Analysis of 2,100 expressed sequence tags (ESTs) in the roots of Rangpur lime (*Citrus limonia* Osbeck) subjected to osmotic stress resulted in the identification of genes involved in the water stress response, including those encoding aquaporins, dehydrins, sucrose synthase and enzymes related to the synthesis of proline [6]. Using a microarray containing 6,000 genes, Gimeno et al*.* [7] investigated the response of the transcriptome of ‘Clementine’ mandarin (*C. clementina* Ex Tanaka) grafted onto ‘Cleopatra’ mandarin (*C. reshni* hort. Ex Tanaka) to water deficit conditions. As observed in other species, genes encoding proteins involved in lysine, proline and raffinose catabolism, hydrogen peroxide reduction, vacuolar malate transport, and defense (including osmotins, dehydrins and chaperones) were induced. Analysis of the NAC family of transcription factors resulted in the identification of one member, *CsNAC1*, that was strongly induced by water deficit in the leaves of ‘Cleopatra’ mandarin and Rangpur lime and by salt stress, cold and abscisic acid (ABA) only in the leaves and roots of ‘Cleopatra’ mandarin [8]. In ‘Cleopatra’ mandarin, Xian et al. [9] isolated a gene encoding *CrNCED1*, which is an enzyme involved in ABA synthesis, and produced transgenic plants that constitutively overexpressed this gene. The transgenic lines displayed tolerance to dehydration, drought, salt, and oxidative damage compared with wild-type plants. Furthermore, low levels of reactive oxygen species (H_2_O_2_ and O_2_^−^) were detected in the transgenic plants under salt stress and dehydration.

In addition to studies addressing the effects of water deficit on the transcriptome, proteomic studies have revealed the role of proteins involved in the complex mechanisms underlying the stress responses of plants [4,10]. Indeed, many proteins related to stress defense, detoxification, carbohydrate metabolism and photosynthesis that participate in the process of adaptation and tolerance to stress have been identified [11,12]. In a study that evaluated changes in the leaves of two contrasting populations of *Populus cathayana* in response to water deficit, 40 drought-responsive proteins were identified: several of the proteins showing altered abundance were involved in transcriptional regulation, secondary metabolism, redox homeostasis and stress defense [13]. An investigation of soybean (*Glycine max* L.) roots subjected to short-term water deficit revealed changes in the abundance of proteins involved in carbohydrate and nitrogen metabolism, cellular defense and programmed cell death [14]. Zadražnik et al. [5] identified drought-responsive proteins in the leaves of two bean cultivars with differing responses to drought stress. These proteins are primarily involved in energy metabolism, ATP conversion, photosynthesis, protein synthesis and proteolysis and stress defence. Changes in protein levels in the leaves of ‘Willow leaf’ and ‘Cleopatra’ mandarin plants subjected to salt stress were analyzed by Podda et al. [15]. Significant variations in the abundance of 44 protein spots were detected. These salt-responsive proteins play roles in photosynthetic processes, ROS scavenging, stress defense, and signaling. However, there are few studies of the root proteome. Analysis of the root proteome of wild watermelon (*Citrullus lanatus* sp.) has revealed that proteins involved in root morphogenesis, carbon/nitrogen metabolism, lignin synthesis and molecular chaperones are differentially regulated under drought stress [16].

In the present study, we used proteomic approaches to analyses changes in the protein profiles of the roots of two citrus rootstock cultivars with contrasting responses to water deficit. Proteins showing significantly altered abundance were selected for identification via mass spectrometry and bioinformatics analysis. In Rangpur lime, the abundance of various proteins involved in protein metabolism, the stress response and proteolysis were modulated under water deficit conditions. In contrast, repair-related proteins contributed more specifically to the response of ‘Sunki Maravilha’ mandarin to this stress. This is the first report to examine the effects of water deficit on the abundance of proteins in citrus roots.

## Results

In the present study, root samples of Rangpur lime and ‘Sunki Maravilha’ mandarin collected in a previous study by Neves et al. [17] were used. Considering the soil moisture data from the previous report [17], two sampling points were selected for proteomic analysis as follows: 1) plants grown in soil with moisture ranging from 0.29-0.28 m^3^m^−3^ were defined as ‘control’ plants, whereas 2) the soil moisture for ‘drought-stressed’ plants ranged from 0.15-0.14 m^3^m^−3^. According to a previous report by Neves et al. [17], stomatal resistance is more pronounced in both varieties at the selected drought stress sampling points. In addition, they have reported that the leaf water potential decreases in water-stressed plants, reaching −1.43 MPa and −1.3 MPa in Rangpur lime and ‘Sunki Maravilha’ mandarin, respectively. Interestingly, Rangpur lime shows a higher growth rate when grown under water deficit compared with the rate observed for ‘Sunki Maravilha’. When subjected to water deficit, the leaves and roots of ‘Sunki Maravilha’ display a progressive increase in the ABA concentration. The lower leaf growth rate that has been recorded for ‘Sunki Maravilha’ mandarin may be associated with its greater leaf ABA concentration. In contrast, in Rangpur lime, alternations between high and low ABA concentrations were observed [17].

### Analysis of root protein profiles in response to water deficit

**Figure 1:**
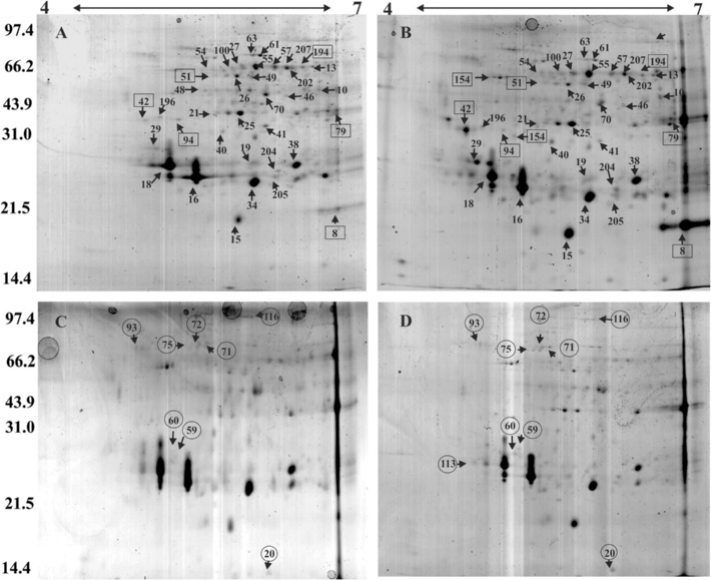
**2-DE analysis of root proteins in Rangpur lime under control conditions (A) and following water deficit (B) and in ‘Sunki Maravilha’ mandarin under control (C) and water deficit conditions (D).** The proteins indicated by the arrows were differentially expressed under the applied treatment. The proteins in the squares are unique to Rangpur lime, and those in the circles are exclusive to ‘Sunki Maravilha’ mandarin.

To elucidate the changes in protein abundance in response to water deficit, comparative analysis of the protein profiles of roots of Rangpur lime and ‘Sunki Maravilha’ mandarin was performed via 2D gel electrophoresis. The root protein profiles of both varieties that were grown under control conditions and subjected to water stress are shown in Figure 1. More than 350 spots were detected in both varieties via image analysis. A total of 81 spots showed significant changes in abundance (*P* < 0.05) in the Rangpur lime roots. These spots were subjected to mass spectrometry (MS) analysis, and 36 proteins were identified. Among these proteins, 11 were increased and 18 were decreased in abundance, and seven proteins were unique to this genotype. In ‘Sunki Maravilha’ mandarin, 72 spots showed significant changes in abundance. Among these spots, 38 proteins were identified, 14 of which increased and 12 of which decreased in abundance, and nine were unique to this genotype.

**Figure 2:**
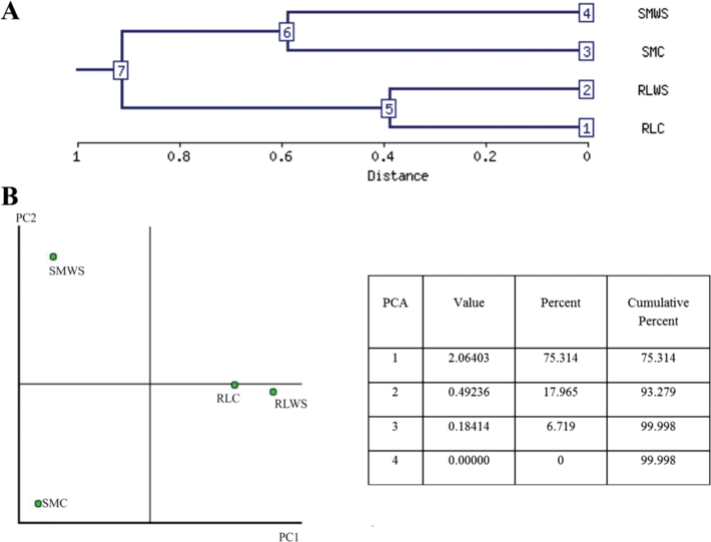
**Principal component analysis (PCA) and evaluation of variance under control conditions and drought in Rangpur lime and ‘Sunki Maravilha’ mandarin. (A)** Hierarchical clustering of the experiments and **(B)** PCA and eigenvalues table in control and water stress-treated samples from Rangpur lime and ‘Sunki Maravilha’.

To understand the relationship between the two plant varieties as a function of water stress, multivariate analysis and principal component analysis (PCA) were performed (Figure 2). PC1 represented 75% of the variance, suggesting that there were differences between Rangpur lime and ‘Sunki Maravilha’ mandarin in response to water deficit. PC2 accounted for 17% of the variance, indicating that in ‘Sunki Maravilha’ , there were differences between the well-watered plants and those under water stress. Interestingly, we observed only minor changes in the protein profiles of Rangpur lime under control versus drought-stressed conditions, suggesting that protein abundance was less affected by water deficit in this variety.

### Identification and analysis of differentially expressed proteins

Spots showing differential intensities under water deficit were excised from the two-dimensional polyacrylamide gel electrophoresis (2D-PAGE) gels and identified via MS (detailed MS/MS results are provided in Additional file 1: Table S1). Some proteins were identified more than once in different spots, reflecting different isoforms, post-translational modifications or alternative mRNA splice forms [18]. Two spots were identified as epidermis-specific secreted glycoprotein EP1-like (8 and 13), five as germin-like (16, 18, 19, 34 and 38), four as 2-phospho-D-glycerate hydrolase (21, 48, 54 and 79), three as mitochondrial processing peptidase alpha-1 subunit (26, 55 and 57), two as putative mitochondrial processing peptidase (202 and 207), four as annexin 1 (51, 59, 60), two as annexin D2 (25 and 94), two as heat shock protein 70 (46 and 154), two as fructokinase (116 and 196) and two as lactoylglutathione lyase (61 and 63).

In addition, some of the proteins that were represented by different spots on the 2D gel showed opposite expression patterns (one spot showed an increase in abundance, whereas the other exhibited a decrease in abundance). The germin-like proteins, which were represented by five spots (16, 18, 19, 34, and 38), and putative mitochondrial processing peptidase (202 and 207) exhibited opposite patterns of accumulation in ‘Sunki Maravilha’ mandarin (Table 1). In contrast, mitochondrial processing peptidase alpha 1 subunit (26, 55, and 57) showed opposite accumulation patterns in Rangpur lime.

**Table 1:** **Identification of differentially expressed proteins in the roots of Rangpur lime and ‘Sunki Maravilha’ mandarin subjected to water deficit**

**ID spot** ^***a***^	**Identified protein reference organism** ^***b***^	**Accession number** ^***c***^	**Mascot score/** ***P*** **-value** ^***d***^	**Mr Theor/Exp** ^***e***^	**pI Theor/Exp** ^***f***^	**Expression level** ^***g***^ **A B C D**	**Fold change (P < 0.05)** ^***h***^ **RL Sk**
8	Epidermis-specific secreted glycoprotein EP1-like *Citrus sinensis*	XP_006477736	155/1e-08	48.8/18	6.26/6.92		−**3.81 np**
10	Dihydrolipoyllysine-residue succinyltransferase component of 2-oxoglutarate dehydrogenase complex 1, mitochondrial-like *Citrus sinensis*	XP_006475040	67/4e-15	51.1/42	9.13/6.55		−2.11 **2.17**
13	epidermis-specific secreted glycoprotein EP1-like *Citrus sinensis*	XP_006477736	130/0.0	48.8/59	6.27/6.61		−1.82 **1.50**
15	miraculin-like protein 1 *Citrus maxima*	AEK31192	176/1e-20	18.9/15	8.18/5.80		**2.32** *1*
16	Germin-like protein subfamily T member 2-like *Citrus sinensis*	XP_006477534	235/2e-169	25.9/24	5.74/5.35		−1.65 **1.2**
18	Germin-like protein subfamily T member 2-like *Citrus sinensis*	XP_006477534	235/2e-169	25.9/24	5.74/5.15		−1.57 -1.38
19	Germin-like protein subfamily T member 2-like *Citrus sinensis*	XP_006477534	235/2e-169	25.9/27	5.74/5.96		−1.39 **2.27**
20	Nucleoside diphosphate kinase *Citrus sinensis*	XP_006464834	60/2e-09	16.3/13	5.91/6.21		Np *1*
21	2-phospho-D-glycerate hydrolase *Citrus trifoliata*	ADD12953	911/2e-18	47.77/39	5.42/5.57		−1.86 **1.56**
25	Annexin D2 *Arabidopsis thaliana*	NP_174810	160/6e-173	36.20/38	5.21/5.83		**1.60 2.47**
26	Mitochondrial processing peptidase subunit alpha-1 *Arabidopsis thaliana*	NP_175610	189/4e-14	48.20/50	6.08/5.79		−1.47 *1*
27	Lipase class 3 family protein *Arabidopsis thaliana*	NP_567515	150/0.0	59.06/59	9.33/5.86		−1.33 **∞**
29	TIR-NBS-LRR type disease resistance protein *Citrus trifoliata*	AAN62351	93/2e-37	41.4/30	7.10/4.91		**3.17** -1.62
34	Germin-like protein 3–3 like *Citrus sinensis*	XP_006477531	222/3e-31	43.3/24	5.73/6.07		*1* -2.79
38	Germin-like protein 3–3 like *Citrus sinensis*	XP_006477531	222/3e-31	43.3/27	5.73/6.45		*1* -3.45
40	Glyoxalase *Theobroma cacao*	XP_007026102	413/4e-144	27.06/33	6.52/5.64		**1.73** -3.26
41	Mitochondrial malate dehydrogenase *Citrus sinensis*	AET22414	285/4e-98	30.89/34	5.2/6.10		−1.71 **∞**
42	Chitinase *Citrus sinensis*	CAA93847	58/0.0045	32.45/35	5.06/4.80		**3.44** np
46	Heat shock protein 70 *Arabidopsis thaliana*	CAA05547	77/0.0	71.4/46	5.14/6.32		**1.64 1.76**
48	2-phospho-D-glycerate hydrolase *Citrus trifoliata*	ADD12953	911/0.0	47.77/46	5.42/5.45		**2.63** *1*
49	Methyl-CPG-binding domain 6 protein *Arabidopsis thaliana*	NP_200746	150/2e-43	24.44/49	9.03/6.0		**1.35 2.07**
51	Annexin 1 *Theobroma cacao*	NP_174810	109/0.0	35.8/48	6.34/5.72		**2.16** np
54	2-phospho-D-glycerate hydrolase *Citrus trifoliata*	ADD12953	911/0.0	47.77/51	5.42/5.7		−1.94 -2.53
55	Mitochondrial processing peptidase subunit alpha-1 *Arabidopsis thaliana*	NP_175610	545/0.0	54.4/56	5.94/5.9		**1.42 ∞**
57	Mitochondrial processing peptidase subunit alpha-1 *Arabidopsis thaliana*	NP_175610	545/0.0	60.79/57	7.06/6.26		**1.83 1.82**
59	Annexin 1 *Theobroma cacao*	EOY16019	566/0.0	35.8/36.1	6.34/5.42		np **2.09**
60	Annexin 1 *Theobroma cacao*	EOY16019	566/0.0	35.8/36.1	6.34/5.42		np **2.38**
61	Lactoylglutathione lyase *Citrus X paradisi*	CAB09799	598/0.0	32.63/66	5.28/6.05		−1.81 **1.66**
63	Lactoylglutathione lyase S-transferase *Ricinus communis*	XP_002518470	52/8e-146	31.5/66	7.63/5.99		−1.90 **1.65**
70	Peroxidase *Citrus maxima*	ABG49115	517/0.0	37.88/44	4.52/6.10		−3.21 *1*
71	Histone ubiquitination proteins group *Populus trichocarpa*	XP_002302510	188/0.0	48.1/67	5.56/5.71		np −3.61
72	Acyl-CoA -N-acetyltransferase *Arabidopsis thaliana*	NP_196882	46.4/7e-29	20.39/22	7.8/5.48		np *1*
75	5-formyltetrahydrofolate cyclo-ligase *Arabidopsis thaliana*	NP_565139	355/1e-119	39.55/39	9.41/6.63		np −2.20
79	2-phospho-D-glycerate hydrolase *Citrus trifoliata*	ADD12953	62.6/0.0	47.78/34	5.54/6.39		−2.68 np
93	mRNA-capping enzyme *Arabidopsis thaliana*	NP_974263	74.4/0.0	78.7/75.57	6.74/5.52		Np *1*
94	Annexin D2 *Citrus sinensis*	CAB09799	116/4e-122	19.8/36	5.30/5.16		−1.79 np
100	ATP synthase beta subunit *Citrus macroptera*	ABM74441	69.4/3e-152	37.07/58	5.01/5.74		−2.68 -4.18
113	2-dehydro-3-deoxyphosphooctonate aldolase *Medicago truncatula*	ABN05924	427/2e-13	31.9/28	6.61/4.91		np **∞**
116	Fructokinase *Oryza sativa*	A2WXV8	70/1e-34	30.3/87	5.50/6.19		np **∞**
154	Heat shock protein-70 cognate protein *Arabidopsis thaliana*	NP_176036	73/0.0	71.4/65	5.10/5.27		∞ np
194	F-box family protein *Vitis vinifera*	XP_002279122	414/4e-139	47.2/55	9.4/6.39		−1.57 np
196	Fructokinase *Citrus unshiu*	AAS67872	219/2e-71	37.5/36	5.11/4.97		−2.54 **1.64**
202	Putative mitochondrial processing peptidase *Arabidopsis thaliana*	BAE98412	202/0.0	51.53/53	5.71/6.49		**2.88** -1.95
205	Putative L-galactose dehydrogenase *Citrus unshiu*	ADV59927	294/1e-18	37.62/25	6.03/6.23		**1.75 ∞**
207	Putative mitochondrial processing peptidase *Arabidopsis thaliana*	BAE98412	480/0.0	51.53/55	5.71/6.33		**1.56 1.47**

^*a*^Spot ID corresponding to the position in the 2D gel illustrated in Figure 1. ^*b*^Protein accession number according to the NCBI database (http://www.ncbi.org). ^*c*^Best matching protein identified by pBLAST analysis of the non-redundant (NCBInr) database. ^*d*^Mascot score *P* value of the homology between citrus proteins and orthologous, homologous, or paralogous proteins, as annotated in NCBInr. ^*e*^Theoretical and experimental masses (KDa) of identified proteins. ^*f*^Theoretical and experimental *pIs* of identified proteins. ^*g*^Expression levels, presented as the % normalised volume, in the control and water deficit-stressed roots. Vertical bars indicate the mean ± SE. **Rangpur lime**: (A) control; and (B) water deficit. **‘Sunki Maravilha’**: (C) control; and (D) water deficit. ^*h*^Fold change (water deficit-treated normalised volume/control normalised volume): **bold** = increased protein abundance; **underlined** = decreased protein abundance; *italics* = no significant difference; np = protein not found in gel; **∞ =** present in one treatment in the genotype.

**Figure 3:**
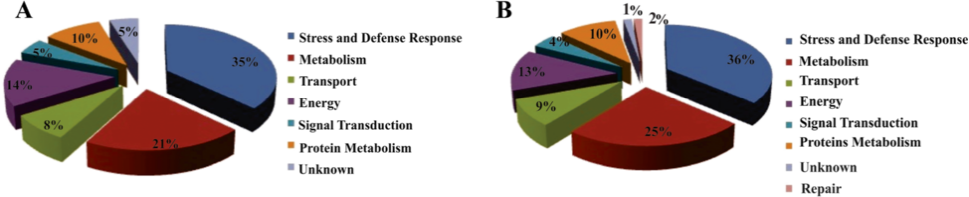
**Functional classifications of drought-responsive proteins. (A)** Functional categorization of proteins that showed significant changes in abundance in Rangpur lime. **(B)** Functional categorization of proteins with significantly altered levels in ‘Sunki Maravilha’ mandarin.

The functions of the identified proteins were inferred using the UniProt database (http://www.uniprot.org). The identified proteins were classified into the following seven major groups according to their possible biological functions: stress and defense response (36% and 35%), metabolism (25% and 21%), transport (9% and 8%), energy (13% and 14%), signal transduction (4% and 5%), protein metabolism (10% and 10%) and unknown (2% and 1%) for Rangpur lime and ‘Sunki Maravilha’ mandarin (Figure 3A and B), respectively. Although the protein groups did not differ significantly between the two studied varieties, an additional class of proteins involved in DNA repair was observed for ‘Sunki Maravilha’ mandarin (Figure 3B).

### Analysis of protein-protein interactions

Analysis of interactomic data of *A. thaliana* orthologous proteins corresponding with the protein interaction profiles of Rangpur lime and ‘Sunki Maravilha’ mandarin (both with and without water deficit) allowed us to draw an interactome network. The network developed for ‘Sunki Maravilha’ mandarin included 723 proteins and 10,430 connectors, and that constructed for Rangpur lime included 566 proteins and 5,954 connectors (Additional file 2: Figure S1 and S2).

Based on analysis of the intersection between the networks of the two varieties, 190 proteins specific to the Rangpur lime network, 347 proteins unique to the ‘Sunki Maravilha’ mandarin network, and 376 proteins shared between the two networks were observed (Additional file 2: Figure S1). The interactome networks obtained for each variety could be divided into several functional clusters. Evaluation of the highly connected regions and gene ontologies of each cluster revealed the presence of 21 clusters in the Rangpur lime interactome network and 22 clusters in the ‘Sunki Maravilha’ mandarin interactome network (Additional file 3: Table S2).

To evaluate the proteins forming the most relevant network, centrality analysis was performed by sorting the proteins into hubs and/or bottlenecks. Among the 45 proteins showing altered abundance in Rangpur lime and ‘Sunki Maravilha’ mandarin, mitochondrial malate dehydrogenase (AT1G53240), mitochondrial processing peptidase (MPPBETA), 2-phospho-D-glycerate hydrolase (LOS2) and nucleoside diphosphate kinase 1 (NDPK1) were considered hubs/bottlenecks. Glyoxalase (ATGLX1), annexin 1 (ANNAT1), glutathione S-transferase (ATG5TF12) and putative L-galactose dehydrogenase (L-Gadh) were only considered to be bottlenecks (Additional file 2: Figure S1A).

### Protein-protein interactions in Rangpur lime

**Figure 4:**
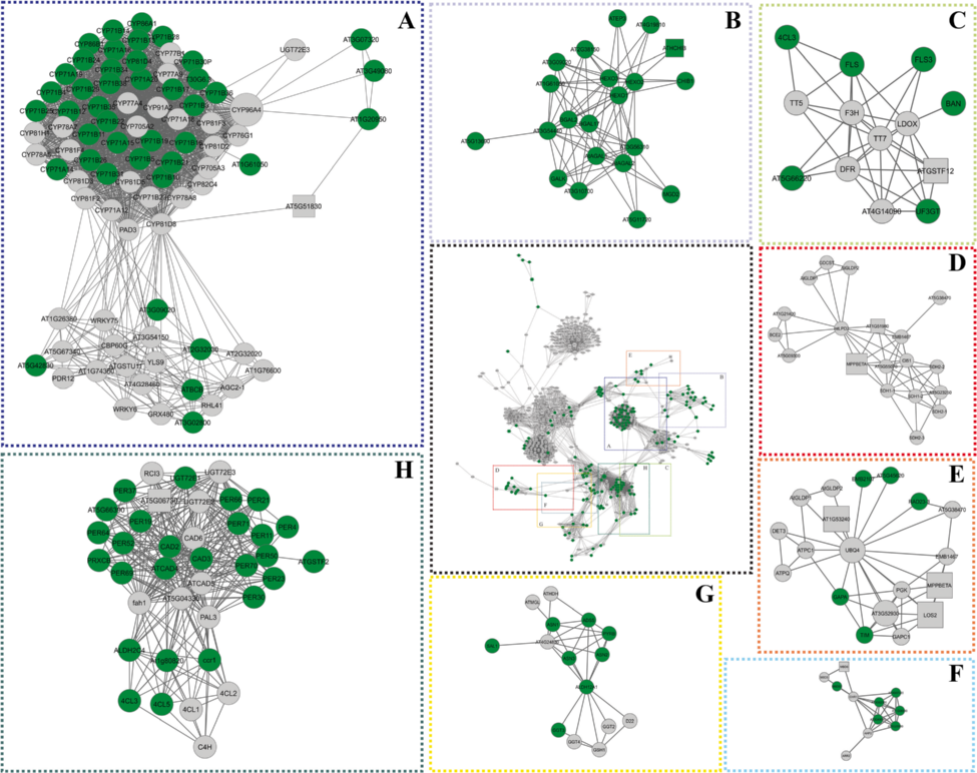
**Interactome network of** ***A. thaliana*** **orthologous proteins related to water stress response of Rangpur lime.** General network with inserts (in colour) that represent clusters (detailed in **A-H**). **(A)** A cluster (in blue) corresponding with proteins related to metabolism, development and the abiotic stress response. **(B)** A cluster (in purple) corresponding with proteins involved in carbohydrate metabolism and systemic responses that are dependent on ethylene. **(C)** A cluster (in light green) containing proteins related to protein modification. **(D)** A cluster (in red) of proteins involved in the response to drought stress. **(E)** A cluster (in orange) corresponding with proteins related to DNA methylation. **(F)** A cluster (in light blue) related to proteins involved in amino acid metabolism. **(G)** A cluster (in yellow) comprising amino acid precursor proteins. **(H)** A cluster (in dark green) corresponding with proteins related to oxidative stress. The circles indicate proteins involved in biological processes corresponding to the network, and the squares indicate proteins that were also differentially expressed during treatment. The green nodes indicate proteins that were unique to the Rangpur lime.

Among the clusters of *A. thaliana* orthologous proteins corresponding with the differentially abundant proteins identified in the two studied citrus varieties, eight were exclusively related to Rangpur lime (Figure 4, clusters A-H). Fructokinase, which was a bottleneck protein in Rangpur lime, was present in a sub-functional network involved in growth, development and the stress response (Figure 4, cluster A, Additional file 3: Table S2) that contained the proteins CYP96A4, CYP71A, CYP70, and CYP76 and representatives of the cytochrome P450 superfamily. This cluster also included WRKY transcription factors (WRKY6 and WRKY75). The WRKY75 transcription factors were associated with osmotin 34 (ATOSM34), which interacted with chitinase (ATHCHIB), which is an enzyme involved in the response to various environmental stresses. Moreover, ATHCHIB was present in the clusters corresponding to metabolism and ethylene-dependent systemic resistance (Figure 4, cluster B) and was related to beta-hexosaminidase (HEXO1), which was in turn associated with galactosidases (BGAL and AtAGAL), which are involved in carbohydrate metabolism. The cluster related to the metabolism of amino acids and protein modification (Figure 4, cluster C) contained cinnamyl alcohol dehydrogenases (CAD2, CAD3, and CAD6), which were linked to numerous peroxidases (Figure 4, cluster H) associated with the oxidative stress response. In the cluster related to the methylation and transposition of DNA, methyl-CpG-binding proteins (MBD6 and MBD3) and chromatin remodelling 1 (CHR1) were found.

Tyrosine aminotransferase 3 (TAT3) was present in a cluster associated with hormone biosynthesis and responses to jasmonic acid and ABA (Figure 4, cluster F), and this protein interacted with coronatine-induced 3 (CORI3), which is involved in methyl jasmonate signalling in guard cells (Figure 4, cluster F). CORI3 was related to superroot 1 (SUR1) and bisphosphate nucleotidase/inositol (SAL1). SAL1 was also associated with a cluster of proteins involved in amino acid metabolism (Figure 4, cluster G) and interacted with proteins involved in the biosynthesis of myoinositol, which is a signalling molecule involved in the stress response (IMPL1, VTC4, MIPS1, and MIPS3), and with phospholipase C (PLC), which is an important protein in the adaptation of plants to environmental stresses. These proteins constituted the cluster related to the response to water deficit stress (Figure 4, cluster D).

### Interactome network analysis for ‘Sunki Maravilha’ mandarin

**Figure 5:**
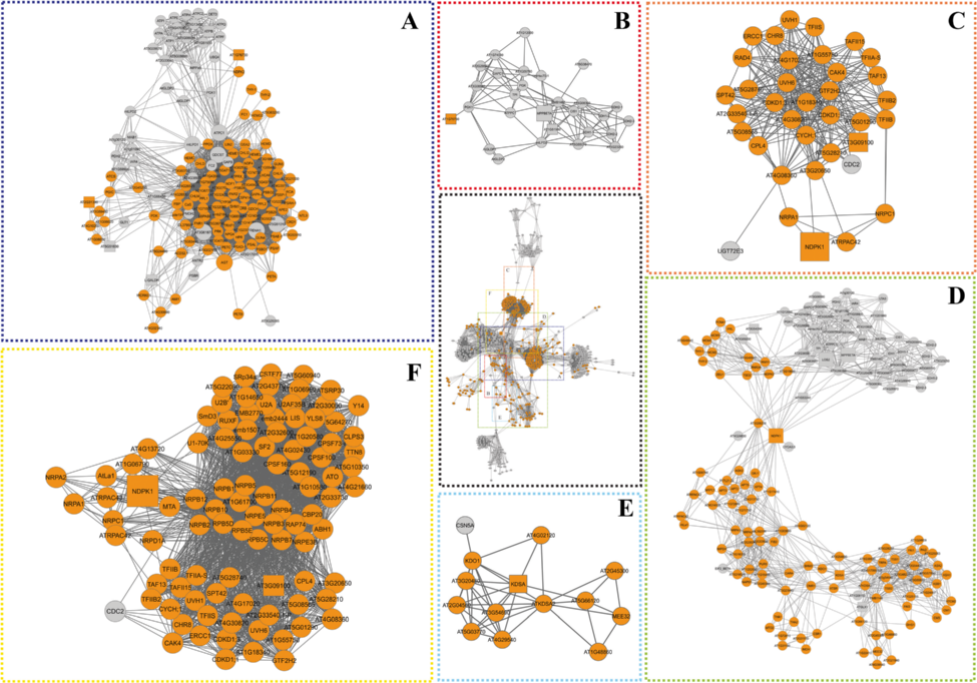
**Interactome network of** ***A. thaliana*** **orthologous proteins related to the water stress response of ‘Sunki Maravilha’ mandarin.** General network with inserts (in colour) that represent clusters (detailed **A**-**F**). **(A)** A cluster (in dark blue) corresponding with proteins related to the metabolism of nucleotides and ubiquitination. **(B)** A cluster (in red) comprising proteins involved in nucleotide metabolism. **(C)** A subgraph (in orange). **(D-F)** Two clusters (in green and yellow in **D** and **F**, respectively) corresponding with proteins involved in DNA repair. **(E)** A cluster (in light blue) containing proteins related to cell division. The circles indicate proteins involved in biological processes corresponding to the network, and the network squares indicate proteins that were also differentially expressed during treatment. The orange nodes indicate proteins that were unique to ‘Sunki Maravilha’ mandarin.

Gene ontology (GO) analysis allowed us to identify the most representative biological processes in the protein interaction network of *A. thaliana* related to ‘Sunki Maravilha’ mandarin (Additional 3: Table S2). The interactome networks were divided into several sub-functional networks (Figure 5), and six of these clusters were found to be unique to ‘Sunki Maravilha’ mandarin orthologous proteins. The main clusters were related to DNA repair and amino acid and nucleic acid metabolism (Figure 5, clusters A-F). A large number of RNA polymerases (NRPB1, NRPB5C, NRPB11, NRPE5, RPB5E and AT1G61700) were found in a cluster corresponding with DNA repair and methylation (Figure 5, cluster F). These proteins were related to the NDPK1 protein, which was exclusive to ‘Sunki Maravilha’ and was considered to be a hub/bottleneck (HB) of this genotype (Additional file 2: Figure S1b). In turn, NDPK1 was associated with LOS2 and MPPBETA (Figure 5, cluster D), which were also considered to be HB proteins in the ‘Sunki Maravilha’ mandarin network (Additional file 2: Figure S1b). This cluster was related to metabolism and biotic and abiotic stresses (Figure 5, cluster D) and included several proteins that showed altered abundance in ‘Sunki Maravilha’ following exposure to water deficit that are known to be involved in the stress response. These proteins included mitochondrial malate dehydrogenase (AT1G53240), glyoxalase (ATGLX1), 2-dehydro-3-deoxyphosphooctonate aldolase (KDSA), fructokinase (AT3G54090) and the dihydrolipoyllysine-residue succinyltransferase component of 2-oxoglutarate dehydrogenase complex 2 (AT4G26910).

The cluster related to DNA repair (Figure 5, cluster C) contained RAD4, 5′-flap endonuclease (ERCC1), chromatin remodelling 8 (CHR8), and ultraviolet hypersensitive (UVH) proteins, which are involved in nucleotide excision from damaged DNA, the regulation of replication and the response to UV light, respectively. A large number of transcription factors were also found (GTF2H2, SPT42, TAF13, TFII-S, TFIIB, TFIIS, and TAFI15). A cluster (Figure 5, cluster E) related to cell division included the following proteins: cytidylyltransferase family protein (KDO1), 3-deoxy-D-manno-octulosonic acid transferase (AT5G03770), 3-deoxy-8-phosphooctulonate synthase (ATKDSA2), and maternal effect embryo arrest 32 (MEE32).

A cluster related to energy, the metabolism of nucleic acids and ubiquitination (Figure 5, cluster A) included an ubiquitin (UBQ4) protein that binds to ATPC1, which was in turn associated with the following proteins: glyceraldehyde 3-phosphate dehydrogenase subunit 2a (GAPA-2), glycine dehydrogenase (GDH), serine transhydroxymethyltransferase 1 (SHM1), A. *thaliana* glycine decarboxylase P-protein 2 (AtGLBP2) and sedoheptulose bisphosphatase (SBPASE), which is involved in the metabolism of osmoprotectants.

## Discussion

Proteins showing altered abundance in response to water deficit in the roots of Rangpur lime and ‘Sunki Maravilha’ mandarin were identified using a proteomic approach. Rangpur lime and ‘Sunki Maravilha’ were selected because in a previous study, these cultivars have been shown to differ in their use of available soil water, ABA accumulation and expression of ABA biosynthesis genes, suggesting that they use different systems to adapt to water restriction [17].

### Protein changes associated with water deficit

Water deficit caused alterations in protein abundance in Rangpur lime and ‘Sunki Maravilha’ mandarin. Approximately 40% of the identified proteins were detected in multiple spots and had different isoelectric points (pIs) and molecular weights (MWs), suggesting the presence of isoforms and post-translational modifications or that these proteins were translated from different products of paralogous genes within a multigene family (Table 1) [19]. The observed changes were related to phenotypic responses that determined the plant tolerance to water deficit [4].

Major changes in protein abundance caused by water stress were observed in ‘Sunki Maravilha’ mandarin compared with Rangpur lime (Figure 2). Under control conditions, Neves et al. [17] have measured higher ABA concentrations in the roots of unstressed ‘Sunki Maravilha’ mandarin compared with Rangpur lime. This finding can be considered to indicate increased physiological responsiveness to biotic and abiotic stresses, which enables better stomatal regulation and consequently reduces water use by ‘Sunki Maravilha’ mandarin plants subjected to water stress. This physiological responsiveness leads to a series of changes at the protein level as an adaptive response to water stress. The proteins identified in Rangpur lime and ‘Sunki Maravilha’ mandarin were functionally categorized in terms of their roles in the response to water restriction. Comparative analysis of protein accumulation in Rangpur lime and ‘Sunki Maravilha’ mandarin, together with the use of a systems biology approach, allowed us to establish a general profile of the biological processes involved in the response to water deficit in these plant varieties (Figure 4). The main functional groups of proteins were examined in relation to water deficit.

### Proteins involved in metabolism and energy

The energy metabolism of proteins is often affected by water deficit. In the present study, the abundance of some enzymes involved in the tricarboxylic acid (TCA) cycle and glycolysis was altered following water deficit in both evaluated varieties. In Rangpur lime and ‘Sunki Maravilha’ mandarin, the mitochondrial malate dehydrogenase levels (spot 41) declined in response to water deficit. This enzyme, which was considered to represent an HB in Rangpur lime (Figure 4A), is a key component of the TCA cycle [20] that is involved in central metabolism and redox homeostasis between organelle compartments [21]. Another protein in the energy metabolism class, ATP synthase, showed decreased abundance in both varieties, which suggested that damage had occurred to the mitochondria and chloroplasts exposed to water deficit. In addition, energy metabolism may have been weakened, which is a disadvantage due to the resulting decreases in the syntheses of ATP and metabolites and feedback signaling. Thus, plants require additional energy to repair the damage caused by water stress.

Mitochondrial processing protein accounted for approximately 11% of the proteome-level changes observed (Table 1), suggesting that mitochondrial function and, hence, plant metabolism were altered and that the integrity of these processes must be protected from oxidative stress induced by drought [22]. Indeed, it is known that a key function of the mitochondria is defense against an excess of ROS. In fact, in plant cells, the mitochondria represent major sources of ROS production and subsequent oxidative damage, as indicated in other proteomic studies [23-25]. These findings suggest that plant tolerance to water deficit may be associated with efficient defense responses against oxidative stress at the cellular and subcellular levels.

LOS2 is an essential glycolytic enzyme that catalyses the interconversion of 2-phosphoglycerate and phosphoenolpyruvate and is induced by several types of abiotic stress, including water deficit and salinity [26]. In the present study, LOS2 showed different patterns of expression and isoforms and was classified as an HB protein in both varieties. In addition, it was identified in a unique ‘Sunki Maravilha’ mandarin cluster that was involved in the stress response and metabolism (Figure 5, cluster D). The opposite expression patterns observed for LOS2 indicate that this protein may play different roles during the water stress response in the two varieties. Systems biology analysis identified interactions between LOS2 and other proteins with important roles in the stress response and energy metabolism, such as NDPK1 (Figure 5, cluster D), which was detected exclusively in ‘Sunki Maravilha’ mandarin and may be involved in the acclimation of this variety to water deficit due to its relationships with the general homeostasis of cellular nucleoside triphosphate [27], oxidative stress responses [28] and water deficit tolerance in bean [5].

### Stress and defense proteins

Approximately 37% of the proteins identified in this study were related to stress and defense (Figure 3). Plants have evolved antioxidant defense pathways to protect cells against the damage caused by high levels of ROS under stress conditions [29]. Several enzymes involved in redox homeostasis were differentially regulated in the responses of Rangpur lime and ‘Sunki Maravilha’ mandarin to water stress, including peroxidase (spot 70, Figure 4, cluster H), lactoylglutathione lyase (spot 61 and 63) and glyoxalase (spot 40, Figure 4, cluster H, Figure 5, cluster D). In the two plant varieties, glyoxalase (Figure 5D) showed opposite abundance patterns compared with those observed for lactoylglutathione lyase. The excessive production of ROS in stressed plants contributes to the accumulation of other toxic compounds, such as methylglyoxal, which is regulated by the glyoxalase system. This system plays a role in tolerance to oxidative stress through the recycling of reduced glutathione (GSH) and specific changes in the absolute concentrations of ROS [5].

Another protein that showed contrasting abundance between the two evaluated varieties was peroxidase, which, together with other enzymes, participates in the removal of H_2_O_2_ from cells. In Rangpur lime, we observed a decrease in peroxidase abundance under water stress conditions, whereas in ‘Sunki Maravilha’ mandarin, the abundance of this enzyme increased. In addition, systems biology analysis identified a cluster related to the oxidative stress response that was unique to Rangpur lime, in which peroxidase was directly associated with phenylalanine, aldehyde dehydrogenase and glutathione S-transferase, which are also involved in the detoxification of ROS (Figure 4, cluster H). The changes in the abundance of several antioxidant enzymes observed in the Rangpur lime and ‘Sunki Maravilha’ mandarin roots may also be necessary to balance the antioxidant system during water deficit. Choi and Hwang [30] have studied the expression of a peroxidase (CaPO_2_) in response to different biotic and abiotic stresses in pepper, demonstrating that its expression is strongly induced by drought, cold, salinity and osmotic stresses. They also have shown that treatment with ABA induces the expression of this enzyme. The increased expression of peroxidase observed in ‘Sunki Maravilha’ mandarin may be associated with the accumulation of ABA, which has been previously observed by Neves et al. [17] during water stress in this variety. Based on our results, it appears that the response of ROS-scavenging enzymes to water deficit in citrus roots is genotype-dependent.

Another protein involved in the stress response is annexin. Two isoforms of this protein with altered abundance following exposure to water stress were identified in our study (ANNAT1 and ANNAT2). The ANNAT1 protein was up-regulated in ‘Sunki Maravilha’ mandarin but not in Rangpur lime, whereas the abundance of the ANNAT2 isoform was altered in both varieties (Figure 5). Annexins are directly involved in the regulation of signaling pathways that are activated by stress, and changes in the abundance of these proteins can alter plant tolerances to various types of abiotic stresses [31,32]. In addition, the protein concentration of annexin in *Arabidopsis* is increased by ABA treatment, and this hormone may be a general regulator of annexin expression in several varieties of plant species [33-35]. Thus, based on the high levels of ABA found in ‘Sunki Maravilha’ mandarin [17], it can be speculated that this protein may function as a positive regulator of the accumulation of annexin in this variety. Moreover, ANNAT1 was classified as a bottleneck protein in ‘Sunki Maravilha’ mandarin (Figure 4B), and it is therefore an important candidate for future experiments designed to provide new insights regarding signaling pathways that are modulated by water stress.

### Repair and processing proteins

Proteins related to the repair and processing of nucleotides, such as mRNA-capping enzyme (spot 93, Figure 5, cluster C and F) and the histone ubiquitination protein group (spot 71, Figure 5, cluster A), demonstrated altered abundance in response to water deficit. These proteins were identified exclusively in ‘Sunki Maravilha’ mandarin, and interestingly, they were present in the interactome sub-network exclusive to this variety. Systems biology analysis identified the interaction of mRNA-capping protein with nucleotide excision repair (NER). The NER pathway is one of the most versatile repair pathways considering the diversity of DNA lesions, including those induced by environmental factors, such as UV radiation [36,37]. DNA repair mechanisms are essential for maintaining genomic stability and integrity under stress conditions, allowing for greater genomic plasticity in response to environmental changes [38].

Protein modification represents a potential target in the engineering of plants with the goal of increasing tolerance to multiple stresses. Post-translational modifications of signaling proteins, such as phosphorylation and ubiquitination, are important for the regulation of gene expression in response to stress. The ubiquitination of proteins can modulate stress response mechanisms involving the regulation and detection of hormone biosynthesis and the control of the abundance of proteins in signaling pathways, particularly transcription factors [39,40].

Studies have shown that ABA is involved in controlling the processing of proteins and RNA [41]. Analyses of the expression of genes in response to ABA treatment in *A. thaliana* have revealed that this hormone alters the expression of ribosomal proteins and genes involved in proteolysis [42]. In addition, Liu and Stone [43] have found that the presence of ABA promotes the self-ubiquitination of multi-domain ubiquitin E3 ligase (KEG) to increase the level of the transcription factor ABSCISIC ACID-INSENSITIVE5 (ABI5) in *A. thaliana*. The high levels of ABA that have been found in ‘Sunki Maravilha’ mandarin by Neves et al. [17] may have induced the post-translational modifications of some proteins that are involved in the response to water restriction, contributing to the improved adaptation to drought of this variety.

### Putative mechanisms in Rangpur lime and ‘Sunki Maravilha’ mandarin in response to water stress

The results of this study demonstrated that the dynamic stress response observed in Rangpur lime included changes in the metabolism of carbohydrates and amino acids as well as alterations in the activation of ROS detoxification and in the abundance of proteins involved in water stress defense (Figures 3 and 4). For instance, L-galactose dehydrogenase (L-GALDH) (spot 205), which exhibited increased abundance in Rangpur lime, held an important position in the network (corresponding to a bottleneck with a high capacity for interacting with other proteins; Figure 4D). This enzyme was directly related to an ascorbate 5’-biphosphate nucleotide (VTC4) that was associated with several proteins in cluster D (such as SAL1 [inositol phosphatase] and PLC1 [phospholipase C]) and is involved in the water deficit response, signal transduction and the response to ABA (Figure 4). SAL1 acts as a negative regulator of drought tolerance in *A. thaliana*, and its inactivation increases the relative water content, improves water-use efficiency, reduces gas exchange and maintains viable tissues during prolonged water stress [44,45]. In addition, PLC1 is strongly induced under various environmental stresses, such as dehydration, salinity, and low temperature, and it plays a role in the inhibition of stomatal opening by ABA [46,47]. We hypothesize that the interactions of these proteins may be related to the faster response of Rangpur to water stress, as demonstrated by its increased soil water collection efficiency. The ABA level increases in the roots of this plant during water deficit conditions in correlation with the activation of defense mechanisms [17].

Protein interaction network analysis suggested that the adaptation of ‘Sunki Maravilha’ plants to stress was aided by the activation of DNA repair and processing proteins (Figure 5). Furthermore, our systems biology analysis revealed that the NDPK1 protein, which was considered to represent an HB in ‘Sunki Maravilha’ mandarin, interacted with proteins in the three clusters involved in the repair and processing of nucleotides (Figure 5, clusters 2, 5 and 6). NDPKs are ubiquitous housekeeping enzymes involved in the response to heat stress [48], UV-B light signaling, growth [49], ROS signaling [27] and phytochrome signaling [50]. Changes in protein abundance and interactions with important proteins involved in DNA repair pathways could be important factors determining the tolerance of ‘Sunki Maravilha’ mandarin to water deficit.

## Conclusions

The present study revealed that the levels of a number of proteins involved in various cellular pathways are affected during water deficit in citrus roots. The results showed that acclimatization to water deficit involved specific responses that differed between Rangpur lime and ‘Sunki Maravilha’ mandarin. Proteins involved in metabolism, energy and signal transduction were down-regulated in ‘Sunki Maravilha’ mandarin. In addition, proteins involved in the repair and processing of nucleotides were identified exclusively in this variety and showed higher levels in the drought-stressed roots compared with the control roots. In contrast, the response to water deficit in Rangpur lime included an increased abundance of proteins involved in transport, protein metabolism, the stress response and proteolysis and a decreased abundance of proteins related to metabolism and energy.

This study provides insights into the effects of drought on the abundance of proteins in the roots of two varieties of citrus rootstocks. In addition, this work allows for a better understanding of the molecular basis of the response to water deficit in citrus. Further analyses are needed to elucidate the behaviors of the key target proteins that appear to be involved in this response.

## Methods

### Plant materials and growth conditions

Rangpur lime (*Citrus limonia* Osbeck) and ‘Sunki Maravilha’ mandarin (*C. sunki* hort. Ex Tanaka) were obtained from the Active Germplasm Bank (BAG) of Embrapa Cassava and Tropical Fruits (Cruz das Almas, Bahia, Brazil). Rootstocks of nucellar and diploid Rangpur lime and ‘Sunki Maravilha’ were used in our proteomics experiments. The varieties were selected according to their contrasting responses to water deficit; Rangpur lime is considered to be more tolerant than ‘Sunki Maravilha’ mandarin [51,17]. We used the same plants that were subjected to water deficit analyses in the study conducted by Neves et al. [17]. The plants were divided into two groups as follows: (i) a control treatment group, in which the plants were constantly irrigated to near field capacity, and (ii) a drought stress group, in which the plants were subjected to a complete suspension of irrigation. The applied water deficit lasted for 40 days, and during this period, soil moisture, transpiration, stomatal conductance and leaf water potential were evaluated. Based on soil moisture data, Neves et al. have selected three sampling periods for plant materials; accordingly, plants with moisture values ranging from 0.29 to 0.28 m^3^ m^−3^ were selected for the control group, and those with values ranging from 0.20 to 0.19 and 0.17 to 0.16 m^3^ m^−3^ were selected for the water deficit group [17]. The relationship between transpiration and soil moisture was estimated for each variety by relating the normalized transpiration rate (NTR) to the fraction of transpirable soil water (FTSW) according to Sinclair and Ludlow [52]. The NTR was calculated by dividing the daily transpiration rate of each plant in the water-deficient group by the average transpiration rate of the control plants of each variety. The FTSW was calculated for each plant daily by subtracting the lower limit of the soil moisture from the soil moisture determined for each plant daily and then dividing that value by the total transpirable soil water for the plant. Rangpur lime seedlings subjected to water deficit displayed decreases in the FTSW and NTR of approximately 20% with respect to the available water (FTSW of 0.19 and NTR of 0.24), and these values in ‘Sunki Maravilha’ mandarin plants were significantly reduced to approximately 30% of the available water (FTSW of 0.33 and NTR of 0.27). The collection of root samples from water-deficient plants was based on the observation of soil water humidity levels of 0.15 to 0.14 m^3^m^−3^. For the control plants, soil water humidity ranged from 0.29-0.28 m^3^m^−3^ [17]. Three independent biological replicates from the drought-stressed plants and control plants of each variety were analyzed. First, the root samples were frozen in liquid nitrogen and stored at −80°C, after which they were lyophilized and stored at −20°C until analysis.

#### Protein extraction

Total root proteins were extracted using the protocol described by Bertolde et al. [53]. The proteins were dissolved in rehydration buffer (7 M urea, 2 M thiourea, 4% CHAPS, 5 mM tributylphosphine, 0.5% IPG buffer 4–7, and a trace amount of bromophenol blue). The amount of protein in each extract was quantified using a 2-D Quant Kit according to the manufacturer's recommendations (GE Healthcare - Brazil).

#### Two-dimensional gel electrophoresis

Approximately 350 μg of protein was dissolved in 250 μl of rehydration buffer and loaded onto 13-cm Immobiline DryStrips, pH 4–7 (GE Healthcare). The proteins were separated in the first dimension using an Ettan IPGPhor II unit (GE Healthcare) with the following program: 500 V for 1 hour, 1,000 V gradient for 1 hour, 8,000 V gradient for 2.5 hours and 8,000 V for 55 min for a total of 80 kV hours. Following isoelectric focusing, the gel strips were equilibrated for 15 min in equilibration buffer containing 7.5 M Tris–HCl, pH 8.8, 6 M urea, 30% (v/v) glycerol, 2% SDS and 1% DDT, followed by another 15 min equilibration with 7.5 M Tris–HCl, pH 8.8, 6 M urea, 29.3% (v/v) glycerol, 2% SDS and 2.5% iodoacetamide. In the second dimension, the proteins were resolved via 12.5% SDS-PAGE using a Ruby SE600 system (GE Healthcare) at 30 mA/gel for 15 min, 40 mA/gel for 30 min, and then 50 mA/gel for 3 hours. After electrophoresis, the 2D gels were stained with 0.08% w/v colloidal Coomassie blue G-250 [54].

#### Image acquisition and statistical analysis

The gels were scanned using an ImageScanner II (Amersham) at 300 dpi, and the images were analysed using ImageMaster 2D Platinum 7.0 software (GE Healthcare). To compare spot quantities between gels accurately, the spot volumes were normalised as percentages of the total volume of all spots in the gel. The normalised percentage volumes (volume %) of the protein spots were then subjected to statistical analysis via analysis of variance (ANOVA) using the web-based NIA array analysis tool [55]. The entire data set was log_2_-transformed and loaded onto NIA, and after determining the biological replicates and log_10_ transformation values, the data were statistically analysed using the following settings: error model “max (average, actual)”, 0.01 for the proportion of the highest variance values removed before variance averaging, 10° of freedom for the Bayesian error model, 0.05 false discovery rate (FDR) threshold and zero permutations. PCA was conducted to assess genotype and experimental differences using the following settings: covariance matrix type, three principal components, 1.5-fold change threshold for clusters, and 0.5 correlation threshold for clusters. The PCA results were represented as biplot graphs, with proteins that exhibited higher tissue expression located in the same area of the graph. A comparison of the abundance of proteins was performed using the following settings: 0.05 FDR and 1.5-fold change threshold.

#### In-gel digestion and mass spectrometry analysis

Selected protein spots were manually excised from the gel and subjected to in-gel trypsin digestion according to Shevchenko et al. [56]. Selected gel plugs were washed extensively with 50% (v/v) acetonitrile to remove dye and SDS impurities. Colourless gel plugs were completely dried with 100% acetonitrile and then vacuum-dried, digested with 25 ng/μl Promega Trypsin Gold (MS grade) in 25 mM ammonium bicarbonate, and incubated overnight at 37°C. The tryptic fragments were eluted from the gel with 50% acetonitrile and 5% formic acid. The extracts were dried under a vacuum to a volume of 15 μl. The resulting peptides from the digests were purified with Reversed-Phase ZipTip C18 pipette tips according to the manufacturer's specifications (Millipore™). The obtained peptides were subjected to online nanoflow liquid chromatography tandem mass spectrometry (LC/MS/MS) using a nanoACQUITY system (Waters, Milford, MA, USA) coupled to a Q-TOF micro mass spectrometer (Waters, Milford, MA, USA). Peptide mixtures were loaded onto a 1.7 μm × 100 mm nanoACQUITY BEH300 column packed with C18 resin (Waters, USA) and separated at a flow rate of 0.6 μl min^−1^ using a linear gradient of up to 50% of solvent B (95% acetonitrile and 0.1% formic acid) over 23 min, followed by an increase to 85% of solvent B in 4 min and maintenance at 85% of solvent B for an additional 3 min. Solvent A was 0.1% formic acid in water. The eluent from the high-performance liquid chromatography (HPLC) column was directly electrosprayed into the mass spectrometer, which was operated in data-dependent acquisition mode to switch automatically between full-scan MS and MS/MS acquisition. The MS and MS/MS raw data were processed using ProteinLynx Global server v2.3 (Waters), and the resulting pkl files were subjected to searches against the NCBI non-redundant (NCBInr) database with the taxonomy parameter set to green plants, using the Mascot server v2.4 (http://www.matrixscience.com). The applied search criteria were as follows: trypsin digestion, carbamidomethyl (Cys) as a fixed modification and oxidation (Met) as a variable modification, a maximum of one missed cleavage event and peptide mass tolerances of ± 0.3 Da for the parent ion and 0.10 Da for the fragment ions. Ion scores of greater than 44 were considered significant (P < 0.05).

#### Systems biology analysis

To obtain information about protein-protein interactions (PPIs) based on the proteomic profiles of Rangpur lime and ‘Sunki Maravilha’ mandarin, we searched for orthologous proteins in *Arabidopsis thaliana*. To achieve this goal, a list of 45 proteins (including 9 proteins exclusive to ‘Sunki Maravilha’ mandarin and 7 exclusive to Rangpur lime) was used to perform reciprocal BLASTp searches [http://blast.ncbi.nlm.nih.gov], after which the initial networks of orthologous proteins of *A. thaliana* were prospected in STRING 9.05 [57]. Using this online search tool, PPIs were downloaded based on the following parameters: no more than 50 interactions, a high confidence score (0.400), and a network depth equal to 2. All of the active prediction methods were enabled, excluding text mining. The true ortholog sequences were obtained in the *A. thaliana* by performing best-reciprocal BLASTP hits from the in silico translation of *Citrus sinensis* and *Citrus clementine* peptides sequences .The BLAST analyses were performed locally on LINUX operating system using the model *C. sinensis* and *C. clementine* databanks extracted from NCBI, the filter soft option (-F "m S") with the local Smith–Waterman alignment algorithm (-s T) and 10- as cut-off e-value. The best-reciprocal BLASTP and the true ortholog identification were supported by the development of a specific script in PEARL programming language (Additional file 4: Table S3).

The interactome networks obtained from the orthologous proteins of *A. thaliana* for each variety were combined in the Rangpur lime network and ‘Sunki Maravilha’ mandarin network using the union function in the Cytoscape 2.8.2 [58] Advanced Merge Network plugin. The Rangpur lime and ‘Sunki Maravilha’ mandarin networks were then analyzed using the Cytoscape 2.8.2 Molecular Complex Detection (MCODE) plugin [59] to detect modules/clusters (densely connected regions) that were suggestive of functional protein complexes. The parameters used for MCODE to generate the clusters were as follows: loops included, degree cut-off of 2, deletion of single connected nodes from the cluster (haircut option enabled), expansion of the cluster by one neighbor shell (fluff option enabled), node density cut-off of 0.1, node score cut-off of 0.2, k-core of 2, and maximum depth of the network equal to 100.

To demonstrate the overlaps of nodes and exclusive regions between the Rangpur lime and ‘Sunki Maravilha’ mandarin networks, the Cytoscape 3.0 plugin Venn and Euler diagrams [60] was used. The major biological processes associated with the clusters generated using MCODE were analyzed with the Cytoscape 2.8.2 Biological Network Gene Ontology program (BiNGO) plugin [61]. The degree of functional enrichment for a given cluster and category was quantitatively computed (*p* value) using hypergeometric distribution, and multiple test corrections were assessed by applying the FDR algorithm [62], which was fully implemented using the BiNGO software at a significance level of P < 0.05. Node centrality analysis was computed using the Cytoscape 2.8.2 plugin Centiscape 1.2 [63] to identify nodes (proteins) with central positions within the networks. The implemented centralities were degree and betweenness. Nodes with relatively higher degrees were termed hubs, and those with higher betweenness were termed bottlenecks. Hubs were nodes that were highly connected, whereas bottlenecks were nodes with a higher probability of joining different clusters [64,65]. HB nodes can be considered to be key regulators of biological processes and to be essential for successful information transfer throughout the network. Furthermore, non-hub/bottleneck (NH-B) nodes were also identified due to the essential nature of the bottleneck nodes in the network dynamics.

Edge centrality analysis was performed using the WERW-Kpath algorithm [66], and data input generation was added as a network attribute in Cytoscape 2.8.2. In addition, quantitative protein expression data were distributed in the protein interaction networks, which included data input as a network attribute. Three networks were generated based on the overlap between the functional and interaction information. The first network compared the proteomic profile of Rangpur lime under water stress (e.g., *treatment a*) to the Rangpur lime control (e.g., *treatment b*), the second compared the proteomic profile of ‘Sunki Maravilha’ under water stress (e.g., *treatment a*) to the ‘Sunki Maravilha’ control (e.g., *treatment b*), and the third compared the proteomic profile of Rangpur lime under water stress (e.g., *treatment a*) to ‘Sunki Maravilha’ under water stress (e.g., *treatment b*), using the color gradient Z = a/(a + b) [67].

### Availability of supporting data

The data sets supporting the results of this article are included within the article and its additional files.

## Additional files

Additional 1: Table S1. Detailed MS/MS results of 46 differentially expressed proteins in *Citrus*. Additional 2: Figure S1. Interactome network of protein involved in water stress response in ‘Rangpur’ lime and Sunki ‘Maravilha’ mandarin. **Figure S2.** Distribution of the quantitative differential expression of proteins in water stress conditions, the interactomic in Rangpur lime and Sunki ‘Maravilha’ mandarin. Additional 3: Table S2. Specific Gene Ontology (GO) classes derived from protein-protein interactions observed in different clusters. Additional 4: Table S3. IDs orthologous proteins *Arabidopsis thaliana-Citrus*.

## Abbreviations

ROS: Reactive oxygen species
LC/MS/MS: Liquid nanoflow chromatography tandem mass spectrometry
PCA: Principal component analyses
ABA: Abscisic acid
MW: Molecular weight
TCA: Tricarboxylic acid cycle
NER: Nucleotide excision repair
NTR: Normalized transpiration soil water
FTSW: Fraction of transpirable soil water
DTT: Dithiothreitol
SDS: Sodium dodecyl sulfate
FDR: False discovery rate
HPLC: High-performance liquid chromatography
PPI: Protein-protein interaction
