# Supplementary material for: Comparative study of the protein profiles of Sunki mandarin and Rangpur lime plants in response to water deficit
Source: BMC Plant Biol. 2015 Mar 3;15:69. doi: 10.1186/s12870-015-0416-6 (PMC4355367; doi:10.1186/s12870-015-0416-6)
Supplement: Additional 1: Table S1. — Detailed MS/MS results of 46 differentially expressed proteins in Citrus. [file 12870_2015_416_MOESM1_ESM.doc]

**Supplementary**

**Table S1**

**Sequence data of 46 differentially expressed proteins in Citrus**

**Proteins have been analyzed by two-dimensional electrophoresis and identified by LC-MS/MS**

***Legend:***

**Spot N°, spot label on the reference map presented in Figure 1;**

**Protein accession number, accession number in NCBI database. For protein identification, a database search was performed on pBLAST against NCBI Viridiplantae to search the best matching protein.**

**Protein name**, **orthologous identified protein names;**

**Reference organism, organism in which the best matching protein has been identified;**

**% Cov, percentage of sequence coverage obtained of identified peptides respect to the length of the orthologous protein;**

**Score, a minimum of two different peptides was used except for three proteins which were characterized by only one peptide, this peptide must contain at least five consecutive fragments and yield a score ≥ 44.**

**Number peptide. number of queries selected to search protein.**

**Peptide change, score of each peptide identified in a spot.**

**Peptide Sequences, list of peptides identified with Mascot software**

| **Spot Nº** | **Protein Acession Number** | **Protein Name**  **Reference Organism** | **% Cov** | **Mascot**  **Score** | **Peptide Number** | **Peptide**  **Score** | **Peptide Change** | **Peptide Sequences** |
| --- | --- | --- | --- | --- | --- | --- | --- | --- |
| **8** | **XP_006477736** | epidermis-specific secreted glycoprotein EP1-like  *Citrus sinensis* | 12 | 120 | 4 | 28  26  42  24 | 2+  2+  2+  2+ | K.FVNEGEFGPFINEYDADYR.M  R.VGGVTK.L  K.ENVDGPYSFVMESDR.L  R.IAAFHYYKVEGVDHYISK.Y |
| **10** | **XP_006475040** | Dihydrolipoyllysine-residue succinyltransferase componente of 2-oxoglutarate dehydrogenase complex 1, mitochondrial-like  *Citrus sinensis* | 4 | 222 | 7 | 34  35  26  22  35  38  32 | 3+  2+  2+  2+  2+  2+  2+ | K.SGEGVAHVAPSEKIPEK.A  K.VETVSEKPKAPSPPPPK.R  K.APSPPPPKR.T  R.VPMTRLR.K  R.SEYKDAFLEK.H  R.EAVFFLRR.I  R.IKDVVEEPR.R |
| **13** | **XP_006477736** | epidermis-specific secreted glycoprotein EP1-like  *Citrus sinensis* | 9.15 | 105 | 2 | 48  57 | 2+  2+ | K.FVNEGEFGPYVNEYDANYR.M  R.CWIAYDLKTLTK.F |
| **15** | **AEK31192** | miraculin-like protein 1  *Citrus maxima* | 15 | 236 | 5 | 27  34  50  44  81 | 2+  2+  2+  2+  2+ | K.VEASRDYYLVSAIR.G  R.GRNELCPLDVVQLSSDSER.G  R.FSMHDK.T  K.TSIINEDVDLNVR.F  R.VDSYDPSR.G |
| **16** | **XP_006477534** | Germin-like protein subfamily T member 2-like  *Citrus sinensis* | 13 | 247 | 4 | 39  76  48  84 | +2  +2  +2  +2 | | K.DPTTFDVFK.R | | --- | | K.DPTTFDVFKR.A | | R.GTEAGIVTR.G | | R.VGDDVINAIR.A | |
| **18** | **XP_006477534** | Germin-like protein subfamily T member 2-like  *Citrus sinensis* | 23 | 235 | 6 | 36  48  28  30  37  56 | 2+  2+  2+  2+  2+  2+ | K.DPTTFDVFK.R  K.DPTTFDVFKR.A  R.AVTFGDVFGFPAVNTQGVALFR.I  R.GTEAGIVTR.G  K.VFLIPR.A  R.VGDDVINTIR.A |
| **19** | **XP_006477534** | Germin-like protein subfamily T member 2-like  *Citrus sinensis* | 25.5 | 426 | 7 | 75  74  56  74  65  44  38 | 2+  2+  2+  2+  2+  2+  2+ | K.DPTTFDVFK.R  K.DPTTFDVFKR.A  R.ATEAGIVLK.G  K.VLTPGMVFLIPR.A  K.SFLVSDDVINAIR.S  R.SARTSNK.S  R.TSNKSGHQFNTR.S |
| **20** | **XP_006464834** | Nucleoside diphosphate kinase  *Citrus sinensis* | 15.3 | 60 | 1 | 42 | 2+ | R.TIIGATNPAQSAPGTIR.G |
| **21** | **ADD12953** | 2-phospho-D-glycerate hydrolase  *Citrus trifoliata* | 15 | 231 | 5 | 23  32  19  81  76 | 2+  2+  2+  2+  2+ | K.HIAELSGNK.N  K.MGVEVYHHLK.A  K.ISGDALKDLYK.S  R.SGETEDTFIADLSVGLATGQIK.T  R.IEEELGAEAVYAGAK.F |
| **25** | **NP_174810** | Annexin D2  *Arabidopsis thaliana* | 18 | 239 | 7 | 48  33  46  23  51  20  18 | 2+  2+  2+  2+  2+  2+  2+ | K.SLDKELTNDFER.V  R.DAFLANEATKR.W  R.KLLLPLVTAYR.Y  K.LLLPLVTAYR.Y  R.YEGDEVNITLAK.S  K.MLLALLGHGDA  K.MLLALLGHGDA |
| **26** | **NP_175610** | Mitochondrial-processing peptidase subunit alpha-1  *Arabidopsis thaliana* | 16.7 | 340 | 7 | 47  70  60  55  32  50  26 | 2+  2+  2+  2+  2+  2+  2+ | R.EVEAIGGNVQASASR.E  R.EVEAIGGNVQASASREQMGYSFDALK.T  R.EQMGYSFDALK.T  K.TYVPEMVELLIDCVR.N  K.SVYTGGDYR.C  K.SAILMNLESR.M  R.MVVSEDIGR.Q |
| **27** | **NP_567515** | Lipase class 3 family protein  *Arabidopsis thaliana* | 10 | 325 | 8 | 66  54  21  20  48  52  27  37 | 2+  2+  1+  1+  2+  2+  2+  2+ | K.GMVLEGLK.R  K.GMVLEGLKR.F  R.NNLGSR.W  R.EVVR.Y  K.SLYATSSVGLPK.W  K.QSKVESGFLSLYNTR.G  K.VESGFLSLYNTR.G  R.RLMELYK.G |
| **29** | **AAN62351** | NBS-LRR type disease resistance  protein  *Citrus trifoliate* | 9 | 93 | 2 | 54  39 | 2+  2+ | K.WPTGSYGDSFLK.L  K.LLHLDLEDCK.S |
| **34** | **XP_006477531** | Germin-like protein 3-3 like  *Citrus sinensis* | 19 | 273 | 6 | 47  32  16  79  19  80 | 2+  2+  2+  2+  2+  2+ | K.DPTTFDVFK.R  K.DPTTFDVFKR.A  R.ATEAGIVLK.G  K.VLTPGMVFLIPR.A  K.VLTPGMVFLIPR.A  K.SFLVSDDVINAIR.S |
| **38** | **XP_006477531** | Germin-like protein 3-3 like  *Citrus sinensis* | 19 | 380 | 7 | 75  48  20  80  39  42  76 | 2+  2+  2+  2+  2+  2+  2+ | K.DPTTFDVFK.R  K.DPTTFDVFKR.A  R.ATEAGIVLK.G  K.VLTPGMVFLIPR.A  K.VLTPGMVFLIPR.A  R.AHVHFQLNVGK.G  K.SFLVSDDVINAIR.S |
| **40** | **XP_007026102** | Glyoxalase  *Theobroma cacao* | 7 | 107 | 4 | 23  36  18  30 | 2+  2+  2+  2+ | R.RFLHAVYR.V  R.FLHAVYR.V  R.SIKFYEK.A  R.TVDKPEYK.Y |
| **41** | **AET22414** | Mitochondrial malate dehydrogenase  *Citrus sinensis* | 9.5 | 110 | 4 | 42  23  12  33 | 2+  2+  1+  2+ | K.KLFGVTTLDVVR.A  K.LFGVTTLDVVR.A  K.ALTK.R  R.TQDGGTEVVEAK.A |
| **42** | **CAA93847** | Chitinase  *Citrus sinensis* | 9.4 | 100 | 4 | 33  21  30  16 | 2+  2+  2+  2+ | K.SYAASVPSDAPK.S  K.AMVESFVNKYR.V  K.AMVESFVNKYR.V  K.SFADDFFSMWR.K |
| **46** | **CAA05547** | Heat shock protein 70  *Arabidopsis thaliana* | 6 | 37 | 1 | 37 | 1+ | R.LGLPPR.Q |
| **48** | **ADD12953** | 2-phospho-D-glycerate hydrolase  *Citrus trifoliata* | 16 | 362 | 6 | 23  67  73  48  54  97 | 2+  2+  2+  2+  2+  2+ | K.HIAELSGNK.N  K.MGVEVYHHLKAVIK.K  K.ISGDALKDLYK.S  K.LTSEVGEK.V  R.MSKQAGWGVMASHR.S  R.IEEELGAEAVYAGAK.F |
| **49** | **NP_200746** | Methyl-CPG-binding domain 6 protein  *Arabidopsis thaliana* | 9 | 106 | 2 | 73  33 | 2+  1+ | K.YYFHVASGRR.F  K.KPNIK.A |
| **51** | **NP_174810** | Annexin D1  *Arabidopsis thaliana* | 42 | 257 | 11 | 35  16  18  22  9  32  41  30  28  26 | 2+  2+  2+  2+  2+  2+  2+  1+  2+  2+  2+ | K.SLDKELTNDFER.V  R.DAFLANEATKR.W  R.KLLLPLVTAYR.Y  K.LLLPLVTAYR.Y  R.YEGDEVNITLAK.S  K.VIKDEYQR.R  R.RNSVPLDR.A  R.AVVK.D  R.AVVKDTSGDYEK.M  K.MLLALLGHGDA  K.MLLALLGHGDA |
| **54** | **ADD12953**  **ABM74441** | 2-phospho-D-glycerate hydrolase  *Citrus trifoliata*  ATP synthase beta subunit  *Citrus macroptera* | 21.3  36.5 | 441  595 | 10  11 | 24  36  67  48  93  50  27  44  52  50  73  68  39  21  42  41  53  65  93  50 | 2+  2+  3+  2+  2+  2+  2+  2+  2+  2+  2+  2+  2+  3+  1+  2+  2+  2+  2+  2+  2+ | K.KIPLYK.H  K.HIAELSGNK.N  K.EAMKMGVEVYHHLK.A  K.MGVEVYHHLK.A  K.MGVEVYHHLK.A  K.VVIGMDVAASEFYGSDK.T  K.ISGDALKDLYK.S  K.VNQIGSVTESIEAVR.M  R.IEEELGAEAVYAGAK.F  K.FRAPVEPY  R.LVLEVAQHMGEGVVR.T  R.TIAMDGTEGLVR.G  R.VLNTGSPITVPVGR.V  R.IMNVIGEPIDEKGDLK.T  K.GDLK.T  K.VVDLLAPYQR.G  K.IGLFGGAGVGK.T  K.TVLIMELINNVAK.A  K.AHGGFSVFAGVGER.T  R.TREGNDLYR.E  R.VGLTGLTVAEHFR.D |
| **55** | **NP_175610** | Mitochondrial-processing peptidase subunit alpha-1  *Arabidopsis thaliana* | 15.7 | 225 | 7 | 50  62  37  29  27  20 | 2+  2+  2+  2+  2+  2+ | R.EVEAIGGNVQASASR.E  R.EVEAIGGNVQASASREQMGYSFDALK.T  R.EQMGYSFDALK.T  K.SVYTGGDYR.C  K.SAILMNLESR.M  R.MVVSEDIGR.Q |
| **57** | **NP_175610** | Mitochondrial-processing peptidase subunit alpha-1  *Arabidopsis thaliana* | 10.3 | 134 | 3 | 44  61  29 | 2+  2+  2+ | K.YAEHVSSAVIFGR.E  K.VASMYSDVLSATILDIEK.C  K.YDMGGAAAVLGAAK.A |
| **59** | **EOY6019** | Annexin 1  *Theobroma cacao* | 34.1 | 235 | 11 | 13  12  22  42  52  13  15  18  18  9  21 | 2+  2+  2+  2+  2+  2+  2+  2+  2+  2+  2+ | M.STLTVPAQVPSVTEDCEQLR.K  K.SLDKELTNDFER.V  R.DAFLANEATKR.W  K.LLLPLVTAYR.Y  K.LLLPLVTAYRYEGDEVNITLAK.S  R.YEGDEVNITLAK.S  R.VVTTRAEVDLK.V  R.RNSVPLDR.A  R.AVVKDTSGDYEK.M  K.DTSGDYEKMLLALLGHGDA  K.MLLALLGHGDA |
| **60** | **EOY6019** | Annexin 1  *Theobroma cacao* | 31.6 | 366 | 11 | 40  39  46  42  72  13  30  16  26  18  13  11 | 2+  2+  2+  2+  3+  2+  2+  2+  2+  2+  2+  2+ | K.SLDKELTNDFER.V  K.SLEEDVGYHTNGDFR.K  R.KLLLPLVTAYR.Y  K.LLLPLVTAYR.Y  K.LLLPLVTAYRYEGDEVNITLAK.S  R.YEGDEVNITLAK.S  R.VVTTRAEVDLK.V  K.VIKDEYQR.R  R.RNSVPLDR.A  R.AVVKDTSGDYEK.M  K.MLLALLGHGDA  K.MLLALLGHGDA |
| **61** | **CAB09799** | Lactoylglutathione lyase  *Citrus X paradisi* | 35.3 | 256 | 11 | 27  20  20  19  22  32  44  19  18  17  18 | 2+  2+  2+  2+  2+  2+  2+  2+  2+  2+  2+ | R.FLHAVYR.V  K.FYTECFGMK.L  K.RDVPEEK.Y  K.LVENIR.A  K.GGTTHIAFVK.D  K.DPDGYIFELIQR.G  R.GPTPEPLCQVMLR.V  R.TVDKPEYK.Y  K.AEVVNLVTQELGGK.I  K.TVLVDNEDFLK.E  K.TVLVDNEDFLKELQSE |
| **63** | **XP_002518470** | Lactoylglutathione lyase  *Ricinus communis* | 5 | 52 | 1 | 52 | 1+ | K.SAEVVNLVTQELGGK.I |
| **70** | **ABG49115** | Peroxidase  *Citrus maxima* | 18.6 | 366 | 8 | 41  60  75  64  50  23  18  35 | 2+  2+  2+  2+  2+  2+  2+  3+ | R.EVLQNAFLSDIR.I  R.GFEVVDDMK.A  R.GFEVVDDMKAAVER.A  R.GFEVVDDMKAAVER.A  R.TLANENLPGPNNSLER.L  K.SFVISMIR.M  K.SFVISMIR.M  R.MGNLKPLTGNQGEIR.S |
| **71** | **XP_002302510** | Histone ubiquitination proteins group  *Populus trichocarpa* | 5 | 143 | 2 | 88  55 | 2+  2+ | R.QQPYDSTLKVVNK.S  R.ADVLSLTNVLER.K |
| **72** | **NP_196882** | Acyl-CoA -N-acetyltransferase  *Arabidopsis thaliana* | 5.1 | 46.4 | 1 | 46 | 2+ | K.MYEKLGYVIYR.R |
| **75** | **NP_565139** | 5-formyltetrahydrofolate cyclo-ligase  *Arabidopsis thaliana* | 12.5 | 139 | 4 | 56  36  25  22 | 2+  1+  2+  2+ | K.ARDLMAETSK.R  K.SWKWIIR.K  K.LLTPQPR.L  K.GEGFAELEYGMLR.Y |
| **79** | **ADD12953** | 2-phospho-D-glycerate hydrolase  *Citrus trifoliata* | 17 | 138 | 8 | 19  18  18  18  17  16  17  15 | 2+  2+  2+  2+  2+  2+  2+  2+ | K.KIPLYK.H  K.HIAELSGNK.N  K.MGVEVYHHLK.A  K.MGVEVYHHLK.A  K.VVIGMDVAASEFYGSDK.T  K.ISGDALKDLYK.S  R.IEEELGAEAVYAGAK.F  K.FRAPVEPY |
| **93** | **NP_974263** | mRNA-capping enzyme  *Vitis vinífera* | 7.1 | 81 | 3 | 42  22  17 | 2+  2+  2+ | K.GRDAVPDNASVNNFVYEVTQFLSR.Q  K.YILVHCTHGHNR.T  K.MLEKEVIEPR.N |
| **94** | **CAB09799** | Annexin D2  *Citrus sinensis* | 22 | 116 | 3 | 26  34  56 | 2+  2+  2+ | K.EAGALLSYDPNLR.L  K.VSDVELEFLTGSDK.I  K.LLLVTLGEHGCR.Y |
| **100** | **ABM74441** | ATP synthase beta subunit  *Citrus macroptera* | 27.5 | 540 | 12 | 50  45  40  68  39  69  41  93  13  17  50  15 | 2+  2+  2+  2+  2+  2+  2+  2+  2+  2+  2+  2+ | R.LVLEVAQHMGEGVVR.T  R.TIAMDGTEGLVR.G  R.TIAMDGTEGLVR.G  R.VLNTGSPITVPVGR.V  R.IMNVIGEPIDEK.G  K.TEHYLPIHR.E  K.IGLFGGAGVGK.T  R.TREGNDLYR.E  R.EGNDLYREMIESGVIK.L  R.EMIESGVIK.L  R.VGLTGLTVAEHFR.D  R.FTQANSEVSALLGR.I |
| **113** | **ABN05924** | 2-dehydro-3-deoxyphosphooctonate aldolase  *Medicago truncatula* | 9.1 | 69.4 | 1 | 27 | 2+ | K.LDGGGVASGGLRELIPCIAR.T |
| **116** | **A2WXV8** | Fructokinase  *Oryza sativa* | 15 | 70 | 2 | 47  23 | 2+  2+ | K.APGGAPANVAIAVAR.L  K.EAGALLSYDPNLR.E |
| **154** | **NP_176036** | Heat shock protein-70 cognate protein  *Arabidopsis thaliana* | 13.6 | 73 | 3 | 30  24  19 | 2+  2+  2+ | R.TTPSYVAFTDTER.L  R.TTPSYVAFTDTERLIGDAAKN  R.IINEPTAAAIAYGLDKK.A |
| **194** | **XP_002279122** | F-box family protein  *Vitis vinifera* | 6 | 52 | 2 | 35  17 | 2+  2+ | K.NRLYVYNPFTR.N  R.LYVYNPFTR.N |
| **196** | **AAS67872** | Fructokinase  *Citrus unshiu* | 22.1 | 55 | 2 | 12  43 | 2+  2+ | K.APGGAPANVAIAVAR.L  K.EAGALLSYDPNLR.E |
| **202** | **BAE98412** | Putative mitochondrial processing peptidase  *Arabidopsis thaliana* | 9.8 | 202 | 5 | 30  36  40  22  74 | 2+  2+  2+  2+  2+ | R.ERDVILR.E  R.MVIAASGAVK.H  R.RIPFAELFAR.I  R.IPFAELFAR.I  R.IDSVDASTVKR.V |
| **205** | **ADV59927** | Putative L-galactose dehydrogenase  *Citrus unshiu* | 19.5 | 356 | 8 | 35  61  46  29  70  62  33  20 | 2+  2+  2+  2+  2+  2+  2+  2+ | R.ERVELATK.F  K.VPIEITIGELK.K  K.VPIEITIGELKK.L  K.LVEEGKIK.Y  K.YIGLSEASASTIR.R  K.YIGLSEASASTIRR.A  R.DAEAEIVPTCR.E  R.ELGIGIVAYSPLGR.G |
| **207** | **BAE98412** | Putative mitochondrial processing peptidase  *Arabidopsis thaliana* | 15.2 | 440 | 9 | 28  30  70  54  60  46  40  38  74 | 1+  2+  2+  2+  2+  2+  2+  2+  2+ | K.RTAR.D  R.ERDVILR.E  R.MVIAASGAVK.H  K.HMGSELAQR.V  K.HMGSELAQR.V  R.VSEADVTR.A  R.RIPFAELFAR.I  R.IPFAELFAR.I  R.IDSVDASTVKR.V |
