# Supplementary material for: Comparative study of the protein profiles of Sunki mandarin and Rangpur lime plants in response to water deficit
Source: BMC Plant Biol. 2015 Mar 3;15:69. doi: 10.1186/s12870-015-0416-6 (PMC4355367; doi:10.1186/s12870-015-0416-6)
Supplement: Additional 2: Figure S1. — Interactome network of protein involved in water stress response in ‘Rangpur’ lime and Sunki ‘Maravilha’ mandarin. Figure S2. Distribution of the quantitative differential expression of proteins in water stress conditions, the interactomic in Rangpur lime and Sunki ‘Maravilha’ mandarin. [file 12870_2015_416_MOESM2_ESM.doc]

**Suplementary material**

**Legends of figures**

**Fig. S1** Interactome network of protein involved in water stress response in ‘Rangpur’ lime and Sunki ‘Maravilha’ mandarin. Venn diagram showing the total number of proteins present in the networks of *A. thaliana* ortologous proteins related ‘Rangpur’ lime and Sunki ‘Maravilha’. Numbers in green correspond to the unique network proteins in Rangpur lime, orange numbers, unique proteins in Sunki ‘Maravilha’. (A) Network clusters featuring exclusive ‘Rangpur’, shown in green. (B) Network with exclusive Sunki ‘Maravilha’ clusters, shown in orange. Differentially expressed proteins are represented by squares and proteins hub-bottlenecks for large circles.

**Fig. S2** Distribution of the quantitative differential expression of proteins in water stress conditions, the interactomic in Rangpur lime and Sunki ‘Maravilha’ mandarin. The color gradient on the graph represents the mapped differential expression genotypes in accordance with the input data (the drought Rangpur-a vs. drought Sunki-b), i.e, express more than b (yellow to red) expressed in most B (blue) and equivalent ab (green). Unique proteins of clusters 'Rangpur' are represented by diamonds, exclusive 'Sunki' represented by hexagons and proteins common to both varieties, by circles.

**
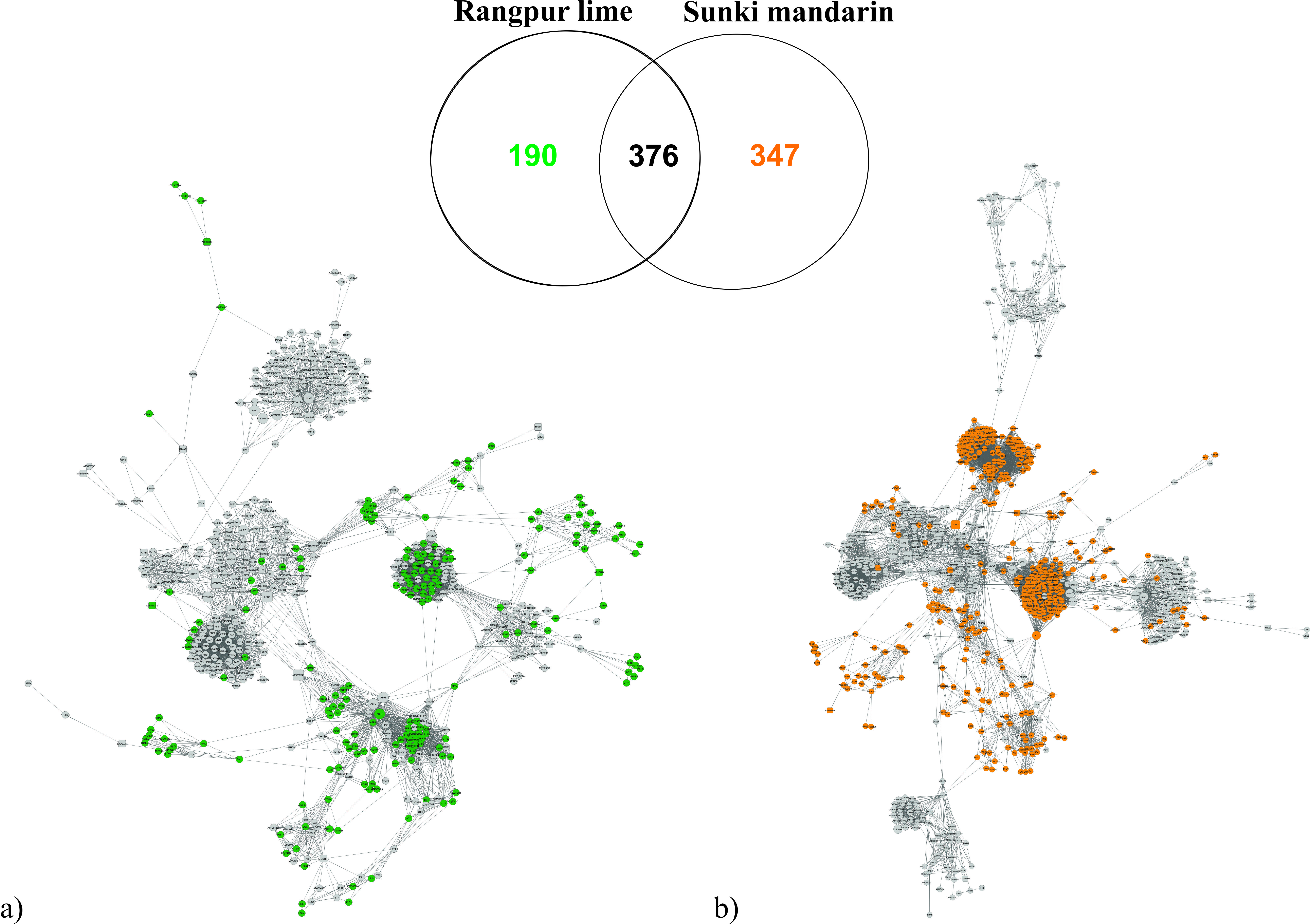
**

**Figure S1**

**
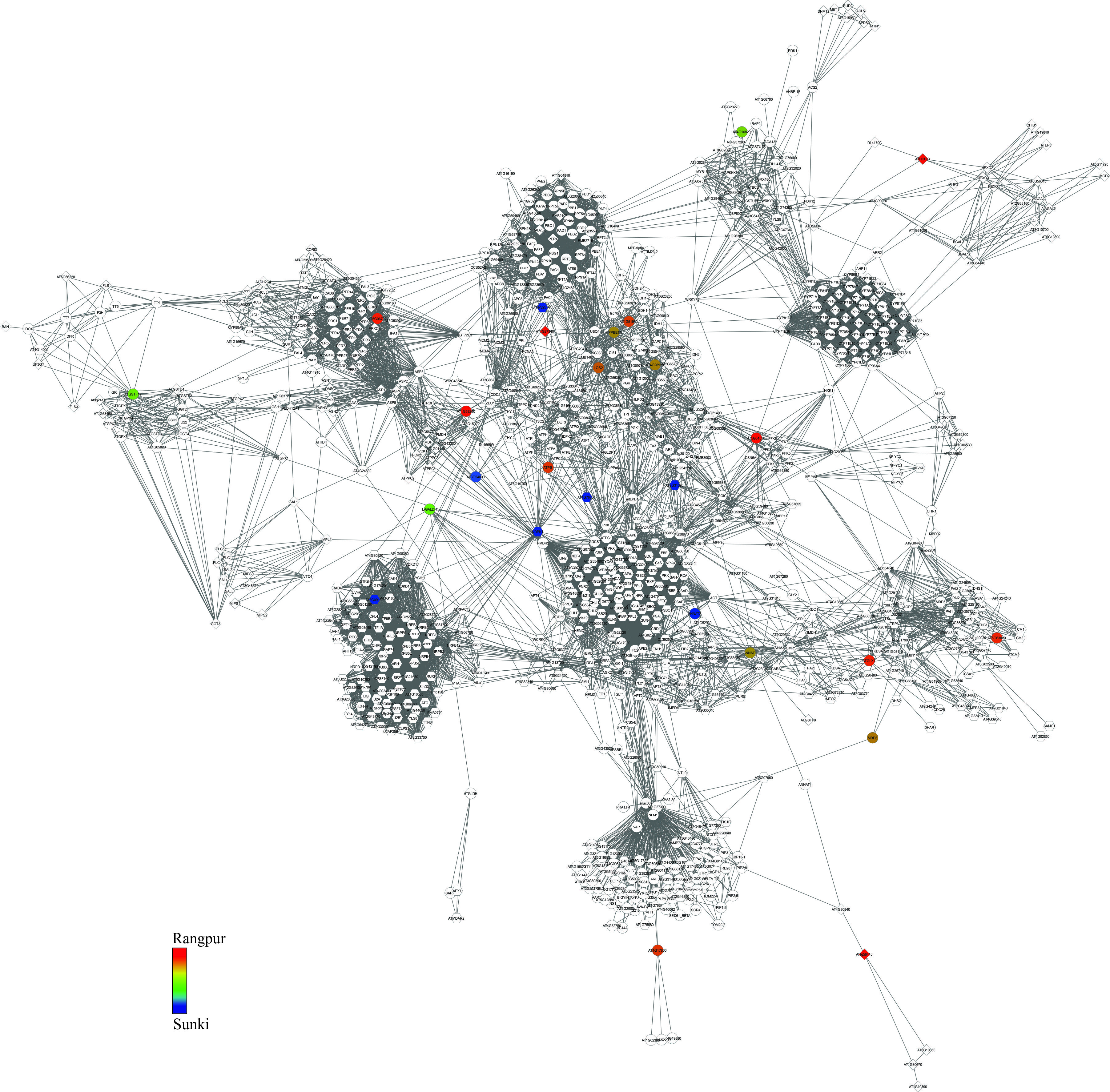
**

**Figure S2**
